# Knowledge on, Attitude towards, and Practice of Sexual and Reproductive Health among Older Adolescent Girls in Bangladesh: An Institution-Based Cross-Sectional Study

**DOI:** 10.3390/ijerph17217720

**Published:** 2020-10-22

**Authors:** Muhammad Zakaria, Farzana Karim, Subarna Mazumder, Feng Cheng, Junfang Xu

**Affiliations:** 1 Department of Communication and Journalism, University of Chittagong, Chittagong 4331, Bangladesh; zakaria@cu.ac.bd (M.Z.); farzanasmail@gmail.com (F.K.); subarna@cu.ac.bd (S.M.); 2 Vanke School of Public Health, Tsinghua University, Beijing 100084, China; 3 Center for Health Policy Studies, School of Public Health, Zhejiang University School of Medicine, Hangzhou 310058, China

**Keywords:** sexual and reproductive health, knowledge, attitude, practice, adolescent girls, Bangladesh

## Abstract

Improving the sexual and reproductive health (SRH) of adolescent girls is one of the primary aims of the Sustainable Development Goals (SDGs). Adequate and accurate knowledge, a favorable attitude, safe behavior, and regular practice contribute to adolescent girls’ SRH, maternal health, and child health. Considering this, this study aims to explore the level of knowledge, attitudes, and practices (KAP) of SRH among college-going older adolescent girls in Chittagong district, Bangladesh. An institution-based cross-sectional study was conducted in four colleges among the older adolescent girl age group of 16–17 years old (*N* = 792) attending a higher secondary grade in Chittagong district. Data were collected using a structured and self-administered questionnaire. Descriptive statistics and multiple linear regression analyses were used to summarize the SRH-related KAP and identify the associated factors, respectively. The level of knowledge about puberty, family planning, maternal health, and HIV/AIDS was not satisfactory among the older adolescent girls. Different myths are common in the rural area with regards to menstruation, which impose several restrictions on adolescent girls and adult women. Standardized coefficients of beta (β) and *p* value < 0.05 in linear regression analyses demonstrated that being a student of the science group (β = 0.29, *p* < 0.001) and reading about or watching SRH issues on media (β = 0.21, *p* < 0.001) were significantly associated with older adolescent girls’ high level of knowledge in this regard. Furthermore, being a student of the science group (β = 0.17, *p* < 0.001), urban residence (β = 0.20, *p* < 0.001), regular SRH communication (at least once a month) with a mother/sister/friend (β = 0.10, *p* = 0.003), and reading or watching any SRH content on media (β = 0.22, *p* < 0.001) appeared as predictors of adolescent girls’ positive attitude towards SRH issues. Moreover, being a student of the science group (β = 0.07, *p* = 0.048), urban residence (β = 0.22, *p* < 0.001), regular SRH discussions with a mother/sister/friend (β = 0.09, *p* = 0.005), pre-knowledge on periods before menarche (β = 0.12, *p* < 0.001), and reading or watching any SRH content on media (β = 0.18, *p* < 0.001) are the most important factors influencing a regular hygienic practice of SRH. This study suggests strengthening SRH-related comprehensive education programs incorporated into the curriculum, the effective use of mass media, and supplying behavioral change communication materials.

## 1. Introduction

Adolescence, a near-universal period of the socialization cycle, is characterized as a phase of human growth and development that occurs after childhood and before adulthood and includes individuals between the ages of 10 and 19 [1]. Adolescence is a transitional stage that includes multidimensional changes, including physical, psychological, emotional, and social changes [2]. Bangladesh has a large adolescent population of approximately 36 million, which means that more than one-fifth of the total population is between the ages of 10 and 19 [3]. According to the population census of 2011, among the adolescent population, about 49% are girls [4]. This population will continue to increase according to population projections [5].

In Bangladesh, the sexual and reproductive health (SRH) status of the adolescent population, including those who are unmarried and married, remains an area of significant concern. Many adolescents, especially adolescent girls in Bangladesh, are not given adequate opportunities to enhance their overall health during their process of growing up [6]. In making informed life choices, they begin to encounter tremendous challenges, for example, a substantial number of adolescents experience risky or unwanted sexual behaviors and do not get prompt or proper care [6]. These issues have caused a high percentage of child marriage practice, adolescent pregnancy, domestic violence, a rising incidence of sexual exploitation, and higher dropout rates due to Bangladesh’s patriarchal social norms [6]. Despite the legal age of marriage for women being 18 years in Bangladesh, a large proportion of marriages still take place before this [7]. The rate of child marriage is still among the world’s highest, with a median age of 16.1 years at first marriage among women [8]. With the highest rate of adolescent fertility, there are 113 live births per 1000 women aged 15–19 years, and 31% of married adolescents aged 15–19 are already mothers or pregnant with their first child, while nearly 70% give birth at 20 years old [9]. Furthermore, adolescent girls also often face a variety of other forms of abuse, including verbal bullying and assault, physical aggression, and sexual exploitation [8]. For example, the first Violence against Women (VAW) survey in Bangladesh of 12,600 women aged 15 and over found that 42% of adolescent girls had experienced violence at the hands of their husband and 11% had experienced violence at the hands of a non-partner, while three-quarters of women had experienced forced sex in adolescence, with 40% having been forced before the age of 15 [10].

SRH is still a cultural taboo in Bangladesh, especially for adolescents, and SRH information and services present a critical gap for unmarried adolescents, particularly girls, which leaves them vulnerable to health risks and discriminatory care [11]. Parents do not feel comfortable discussing SRH issues with their adolescent children, and schools provide minimal information on SRH [12]. Similarly, adolescent boys also face similar educational and cultural restrictions. A dearth of adequate knowledge and appropriate information on SRH makes them confused, scared, excited, and curious; causes insomnia; and raises a number of questions in their mind [13,14,15]. Adolescent boys look for support from their close ones for tackling these problems, but the irony is that no one helps them or even shows enough empathy to respond to their query [16,17]. In Bangladesh, reproductive health is still generally focused on women’s reproductive health concerns. Few SRH programs address males for helping them to get better care for their partners, which may make the SRH situation of older adolescent girls worse, especially for married girls in Bangladesh.

However, Bangladesh has no nationally representative data that measure the level of knowledge, attitudes, and practices (KAP) on SRH among adolescent populations [6]. The BDHS (2014), which merely highlights ever-married adolescents’ knowledge of HIV/AIDS, reporting that only 12% of ever-married adolescents had a comprehensive knowledge in this regard, is further evidence of the low level of knowledge among adolescents about SRH issues [9]. A low level of knowledge due to inaccurate information is often related to negative SRH consequences. However, there is no evidence on the level of SRH-related KAP of adolescent girls in Bangladesh, and little research has been conducted to assess SRH knowledge and awareness in this regard. Therefore, this study aimed to develop knowledge on the SRH-related KAP and associated influencing factors among older adolescent girls in Chittagong district in Bangladesh, which are the basis for, and the first to contribute to, improving the KAP of adolescents in Bangladesh.

## 2. Materials and Methods

### 2.1. Study Design and Population

An institution-based cross-sectional study was conducted from 17 June 2019 to 23 July 2019. Our study population was older adolescent girls aged 16–18 years, who had reached puberty at least two years preceding the survey and were studying at an intermediate level in government/autonomous or private colleges located in Chittagong district.

### 2.2. Sample Size and Sampling Procedures

For selecting the colleges, a probability sampling method was used for urban and rural areas, respectively. For urban participants, two colleges were randomly selected for the study from the lists of two types of colleges of Chittagong City. Similarly, for rural respondents, two colleges were taken from Satkania Upazila (sub-district) following the lottery method. Adolescent girls who attended the selected colleges in the higher secondary classes participated in our study. Finally, 792 respondents (*N* = 792) were incorporated into our study. The mean age of the respondents was 16.59 years (SD = 0.49).

### 2.3. Data Collection and Measures

Data were collected using a structured and self-administered questionnaire in Bengali. Seats were spaced far apart to ensure confidentiality of the participants. Sixty adolescent girl students outside the study area were pretested using the questionnaire. After the pretest, the questionnaire was reviewed for wording appropriateness, clarity of the content, and whether the elicited instructions were accompanied by responses.

The questionnaire included socio-demographic information on students and their parents; reproductive health-related characteristics of the study participants; and their SRH knowledge-, attitude-, and practice-related items. The questions measuring the level of knowledge were based on the basic information mentioned in related literature, textbooks, posters, and brochures published for addressing adolescent girls. The content validity of the questionnaire was reviewed by five experts who worked in the SRH field. All items retained in the scale were reviewed by the experts separately. The reviewers’ identities were not revealed to each other, aside from the researcher. Some changes were made to the questionnaire based on experts’ recommendations. The internal consistency was also measured. Cronbach’s Alpha (α) value was acceptable among 10 attitude-related items (α = 0.78), but low among 10 knowledge-related items (α = 0.55) and 10 practice-related items (α = 0.54), which should be kept in mind when interpreting the results. The response options regarding KAP involved a five-point Likert scale (for the knowledge section: definitely true, probably true, not sure, definitely false, and probably false; for the attitude section: strongly agree, agree, neutral, disagree, and strongly disagree; for the practice section: always, often, sometimes, rare, and never). For the percentage distribution of respondents’ responses regarding KAP, five scales of each section were recoded into three categories because of the low frequency at the endpoint of the scale. In our study, regular SRH communication indicates discussions of adolescent girls with their mothers or sisters at least once in a month that covered safe practices and complexities during menstruation, physical and mental changes during puberty, safe sexual health and behavior, maternal health, pregnancy, and the delivery process, as well as sexual harassment and sexually transmitted diseases (STDs).

### 2.4. Data Analysis

Because 32 respondents (3.88%) were excluded from the analysis due to incompleteness, the final number of participants employed for data analysis was 792. Descriptive statistics with the frequency and the mean with a 95% confidence interval were used to describe the SRH-KAP of older adolescent girls. Multiple linear regression analyses were used to examine respondents’ SRH knowledge, attitude, and practice. The confounding and multicollinearity were checked. Analysis of Variance (ANOVA) values for the overall SRH knowledge (*F* = 17.418, *p* < 0.001), SRH attitude (*F* = 16.757, *p* < 0.001), and SRH practice (*F* = 18.546, *p* < 0.001) showed that our multiple linear regression model performed well and would be a good predictor of the main outcome variables. Variables with *p* < 0.05 were considered statistically significant.

### 2.5. Ethical Approval and Consent to Participate

Written informed consent was obtained from each participant before data collection. The study protocol was reviewed and approved by the Research and Publication Office of the University of Chittagong. The study was conducted in accordance with the Declaration of Helsinki, and ethical approval for the study was provided by the Institutional Review Board for Human Subject Research, Research Centre for Public Health at Tsinghua University (No. THUSM/PHREC/2020400-011).

## 3. Results

### 3.1. Socio-Demographic and Other Descriptive Characteristics of Older Adolescent Girls

Table 1 shows the socio-demographic characteristics of older adolescent girls and their parents. Of older adolescent girls, 458 (57.8%) were from the humanities group, 229 (28.9%) were from the commerce group, and 105 (13.3%) were students of the science group. There was an equal number (396, 50%) of older adolescent girls from urban and rural areas. With regards to religion, the majority of older adolescent girls (90.3%) were Muslim. Three-quarters of the older adolescent girls acknowledged watching TV regularly, and one-fifth reported their Facebook use. The absolute majority of older adolescent girls’ mothers (741, 93.6%) were housewives. More than half of older adolescent girls (58.8%) reported that their mothers watched TV regularly, whereas only 63 (8%) acknowledged their mothers’ use of Facebook.

**Figure 1:**
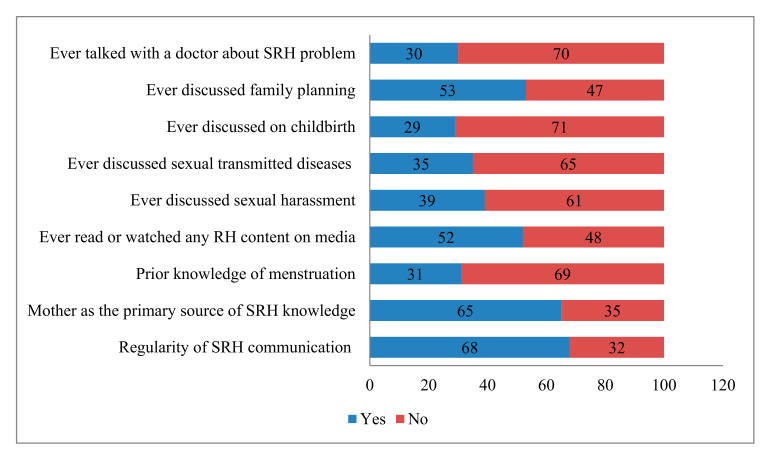
Percentage distribution of older adolescent girls’ sexual and reproductive health (SRH)-related characteristics.

Figure 1 portrays the different SRH-related characteristics of older adolescent girls, reporting that more than two-thirds of them (68.6%) did not have prior knowledge of menarche or menstruation. Most of the adolescent girls (65%) acknowledged their mothers as the primary source of knowledge on SRH. Furthermore, more than two-thirds of the older adolescent girls (68%) had regular (at least once in every month) communication regarding SRH issues with their mothers or sisters.

Moreover, more than half (52%) of older adolescent girls had read or watched SRH content on media, while 30% had talked with a doctor or health worker about SRH matters. Figure 1 also demonstrates different important issues covered in SRH-related discussions with a mother, sister, and other female relatives. The topics discussed in interpersonal communication were family planning (53%), sexual harassment (39%), STIs (35%), and the childbirth process (29%).

### 3.2. Older Adolescent Girls’ SRH Knowledge

Percentage distributions with a mean score of older adolescent girls’ SRH knowledge-related items are reported in Table 2. The study findings depicted that 491 (62%) older adolescent girls had an accurate knowledge of physical and psychological changes occurring in the adolescence period, and 285 (36%) older adolescent girls answered correctly about whether menstruation is a kind of disease. Moreover, only 238 (30%) older adolescent girls had appropriate knowledge in terms of whether taking birth control has any adverse effect on the sexual relationship of a couple. Pertaining to the item, ‘the carrier of the STIs may unintentionally transmit the virus to its partner,’ 434 (54.8%) older adolescent girls had complete information. In addition, 317 (40%) of older adolescent girls responded correctly regarding whether HIV can spread through mosquitoes and fleas, whereas 201 (25.4%) knew that HIV is not spread by an infected person coughing and sneezing.

### 3.3. Older Adolescent Girls’ SRH Attitude

Table 3 displays the percentage distributions of older adolescent girls’ SRH attitude-related items with the mean point. Of the older adolescent girls, 252 (31.8%) correctly agreed that ‘sexual education leads to more sexual activity’. Three-quarters of older adolescent girls thought that a teenage girl can go into the kitchen during her menstrual cycle. Moreover, more than three-quarters (78.7%) of older adolescent girls had the opinion that an adolescent girl can touch anyone during the menstrual period, while more than three-quarters of students expressed that an adolescent girl can go outside and to college during the menstrual period. Furthermore, 615 (77.7%) older adolescent girls thought that adopting the birth control method does not count as a sin.

### 3.4. Older Adolescent Girls’ SRH Practice

Percentage distributions with a mean score of older adolescent girls’ SRH practice-related items are reported in Table 4. More than half of older adolescent girls had a normal behavior with respect to the physical and psychological changes that occurred during the adolescence period, while about two-thirds of the older adolescent girls reported that they use cloth during menstrual periods instead of sanitary pads. About three-quarters of the girls changed their pad or cloth after 5–6 h during their menstrual period. In addition, 484 (61.1%) older adolescent girls reported that they usually felt at ease when talking about sexual and reproductive health with mothers, relatives, and friends, whereas 456 (57.6%) students felt comfortable using the phrase ‘I am sick’ during their menstrual cycle instead of ‘I have a period.’

### 3.5. Linear Regression Analysis Reporting Factors Associated with SRH-Related KAP

The results of the linear regression analysis depicting factors associated with older adolescent girls’ SRH knowledge, attitude, and practice are reported in Table 5. It is clear that being a student of the science group (β = 0.294, *p* < 0.001) and reading about or watching SRH issues on media (β = 0.214, *p* < 0.001) are significantly associated with older adolescent girls’ high level of knowledge in this regard. Furthermore, being a student of the science group (β = 0.169, *p* < 0.001), urban residence (β = 0.203, *p* < 0.001), regular SRH communication with a mother/sister/friend (β = 0.096, *p* = 0.003), having knowledge on periods before experiencing them (β = 0.069, *p* = 0.040), and reading or watching any SRH content on media (β = 0.217, *p* < 0.001) appeared as predictors of older adolescent girls’ positive attitude towards SRH issues. Moreover, it is obvious that being a student of the science group (β = 0.072, *p* = 0.048), urban residence (β = 0.219, *p* < 0.001), mothers’ regular TV watching (β = 0.080, *p* = 0.024), respondents’ regular SRH discussions with a mother/sister/friend (β = 0.090, *p* = 0.005), pre-knowledge on periods before menarche (β = 0.123, *p* < 0.001), students whose primary source of reproductive health was their mother (β = 0.082, *p* = 0.012), reading or watching any SRH content on media (β = 0.180, *p* < 0.001), and visiting and talking with a doctor or health worker about SRH issues (β = 0.080, *p* = 0.016) are the most important factors influencing a regular hygienic practice of SRH of older adolescent girls.

## 4. Discussion

Our study demonstrates that that majority of older adolescent girls did not have prior knowledge of menstruation while experiencing menarche. This finding is consistent with others [18,19]. For example, Bano and Al Sabhan [18] found that 62.5% of girls who studied at a university in Saudi Arabia where religious conservativeness exists, like in Bangladesh, were not aware of this natural phenomenon until menarche, while Hakem et al. [19] revealed that in India, 59.6% of non-government school girls aged 13–19 years knew about the menstrual cycle before menarche, while 48.8% of government school girls had such knowledge. Traditionally, parents in Bangladesh think that pubertal changes, including menstruation, are a natural phase of human development that should remain a secret to adolescents before they experience physical and psychological changes. Usually, mothers think that prior knowledge about sexual health may lead to adolescents becoming sexually active [20], which leads to the majority of adolescent girls not knowing about menstruation. However, adolescents may suffer from fear, depression, and anxiety after experiencing the rapid development of significant biological changes as they had no prior knowledge regarding SRH.

The present study shows that most of the older adolescent girls considered their mothers to be their key informants of SRH issues. Mothers also appeared as the primary source of SRH information. This is because daughters have a trustworthy relationship with their mothers due to gender homogeneity [20]. Similarly, Gaferi et al. [21] also explained the result of good communication between mothers and daughters by the fact that many mothers are being educated nowadays. Furthermore, Kumar and Srivastava [22] claimed that educated Indian mothers are not hesitant to talk about SRH issues, including menstruation, with their daughters.

As was observed, about half of the adolescent girls neither read about nor watched any SRH-related content on media. The lack of perceived importance of the adolescent girls of the rural area may result in a low media exposure of SRH issues. Moreover, less than one-third of the students had ever consulted with health care providers regarding the SRH problem. Inadequate health care services across the country and the tendency to conceal SRH-related problems because of the perception of them being taboo may contribute to this low percentage of access to health care.

This study found that about one-third of the older adolescent girls had inaccurate knowledge regarding puberty health. The absence of open and frequent discussions on this important topic within the family, classroom, and social network; a lack of SRH health campaigns; and inadequate SRH-related content on mass media due to the perceived taboo of the issues have led to the restriction of a steady flow of SRH information and ignorance about adolescence health among the college-going girls. Furthermore, a portion of the study participants were also unaware of family planning and maternal health issues. According to social perception, regardless of their age, women are considered to have matured after their marriage. Therefore, SRH discussions are forbidden for unmarried girls, and significant numbers of female students are uninformed at their most crucial transitional phase of life [11].

Discussions on sexuality and sexually transmitted diseases are prohibited in social spaces in the country. Ideas about HIV/AIDS have been given in an elementary form in textbooks prescribed by educational institutions as these matters are considered taboo. In general, ideas that have been given are that HIV/AIDS can occur if anyone uses a syringe used by an HIV-infected person or uses untested blood, or when a child is born by an infected mother. The most important messages, such as unsafe sexual intercourse, are often left out of discussions. Additionally, empirical evidence revealed that content delivery in school education continues to remain inefficient, and teachers often skip the chapters, or ask students to study them at home [6]. Teachers never utter the word ‘sex’ or avoid it while teaching in the classroom. Because of cultural impediments, everyone has an adverse attitude towards comprehensive sex education. Any curriculum for sex education may be viewed as promoting premarital sex and would therefore be unacceptable to parents [13].

Different myths are common in the rural area with regards to menstruation, creating religious dogmas and cultural orthodoxy that impose restrictions on adolescent girls and adult women. These restrictions prohibit adolescents from going outside, even to school; entering the kitchen; touching any male; brushing their hair; and seeing themselves in the mirror. Some respondents, most of them from rural areas, believed that they should follow these restrictions. Various types of restrictions related to menstruation also exist in other countries, such as India, Nepal, and Saudi Arabia [19,22,23,24,25,26]. The restrictions also include not serving food and attending guests [22,23], food restrictions [21,22,27], religious practices [22,23,24,27], sleeping on the bed or sitting on the sofa [22], and physical exercise [21,22]. In Bangladesh, the conventional practice rooted in the conservative socio-cultural structure has taught women that their desires, dreams, pains, aspirations, sorrows, and joys can never be expressed outside the home or in public. Discussions about the menstruation process of women are thought to be a matter of shame, so unrealistic, unscientific, and superstitious thoughts on this very natural matter take root in the society to a greater extent.

Our study findings also reported that about half of the adolescent girls feel shy and fearful of puberty issues; consequently, they are reluctant to reveal SRH-related problems. In the rural area, mothers still feel uncomfortable while discussing SRH-related matters with their daughters, due to traditional values and conservative attitudes. Therefore, it is not a particular matter for female students to feel discomfort about reproductive health, whereas a very dear one like a mother feels uncomfortable discussing periods. Our study depicts that the majority of adolescent girls used clean cloth during their menstrual cycle, which is very unhealthy because it can cause fungal infections and urine infections. In Bangladesh, the use of sanitary napkins is a very recent trend. As a result of publishing advertisements on media, currently, the level of awareness is gradually increasing. Purchasing capacity also influences the use of a sanitary napkin. Though it is more convenient for a female student who lives in a city, the use of napkins depends on the financial well-being and availability of a student living in a village. However, irrespective of the area of residence, the average use rate of the sanitary pad is higher than the findings of some studies conducted in India [19,24] and lower than that of Saudi Arabia [21]. Gaferi et al. [21] argue that the common use of sanitary napkins may be a result of the high availability and increased awareness from television in this regard.

This study also examined the factors better predicting the KAP of SRH of the study participants. We found that being a student of the science group, urban residence, regular SRH discussions, prior knowledge on SRH, mother as the source of SRH information, ever reading or watching SRH content on mass media, and ever talking with a health professional regarding SRH problems are the significant factors associated with a better status of SRH knowledge, attitude, and practice. Some of these factors are also supported by other studies [28,29].

## 5. Conclusions

This survey among older adolescent girls aged 16–18 years has shown, overall, a higher than average level of sexual and reproductive health knowledge and practice and better status of attitude in this regard. However, knowledge gain, a positive attitude, and regular practices are augmented by factors such as studying in a science group, urban residence, regular SRH communication, and reading or watching SRH-related content on media. Although the college-going study participants had already reached the late adolescence stage, their knowledge regarding menstruation and HIV/AIDS was poor, and their attitude towards sexual education was inappropriate. Furthermore, different myths and misconceptions that should be refuted were common among one-fifth of the adolescent girls. About two-thirds of the respondents used cloth instead of sanitary pads during their menstruation cycle. Moreover, the girls usually felt comfortable expressing themselves as sick, whereas most of them thought of periods as a disease.

This study questions the existing school curriculum and teaching method which are employed to teach SRH education among adolescents and constructs new ground for conducting policy research to develop an apposite strategy for improving the SRH status through cultural appropriateness. This study also suggests conducting studies among adolescent boys to investigate their SRH status.

Given the context, it is crucial to meet the sexual and reproductive health needs and rights of adolescent girls, as a significant number of this cohort are married before the age of 18. These needs can be met by ensuring the provision of quality and age apposite sexuality education commencing with very young adolescents, and the delivery of quality age and gender appropriate SRH information in line with global standards, adapted to suit the present context of Bangladesh. Reproductive and menstrual health and hygiene should be more detailed and comprehensive in the school curriculum. Adolescent girls should receive counseling services at government health facilities and schools. The use of radio, television, and social media could also be an effective strategy for supplying teenagers with information about puberty health, especially those living in remote and rural areas. Sanitary pads should be made available by the government to all adolescent females at a subsidized rate to make them affordable.

### Limitations of the Study

The study participants’ information regarding their knowledge, attitude, and practice of sexual and reproductive health might have been influenced by social desirability, which may have affected the validity of the result. Moreover, Cronbach’s alpha on the knowledge and practice measure is lower than we would have liked, and this measure requires further refinement. Moreover, no qualitative data collection method was used, so this study could not obtain a better explanation and more in-depth insight into such a taboo topic.

## Figures and Tables

**Table 1:** Descriptive characteristics of older adolescent girls in the study (*N* = 792).

Variable	Frequency (*N*)	Percentage (%)
Major group		
Humanities	458	57.8
Commerce	229	28.9
Science	105	13.3
Residence		
Urban	396	50.0
Rural	396	50.0
Religion		
Muslim	715	90.3
Hindu	77	9.7
Buddhist	11	1.4
Family size (persons) *		
3–4	174	22.0
>4	414	78.0
Fathers’ education		
Illiterate	79	10.0
Primary (1–5 grade)	231	29.2
Secondary (6–10 grade)	255	32.2
Higher secondary (11–12 grade)	117	14.8
Bachelor (12+ grade)	110	13.9
Mothers’ education		
Illiterate	72	9.1
Primary (1–5 grade)	248	31.3
Secondary (6–10 grade)	326	41.2
Higher secondary (11–12 grade)	96	12.1
Bachelor (12+ grade)	50	5.3
Number of income holder in the family		
1	613	77.3
>1	179	22.7
Fathers’ occupation		
Agriculture	155	19.6
Expatriate & others	189	23.9
Business	187	23.6
Service	261	33.0
Mothers’ occupation		
Housewife	741	93.6
Others	51	6.4
Watching TV		
Regular	597	75.6
Irregular	193	24.4
Mobile use		
Yes	203	25.6
No	589	74.4
Facebook use		
Yes	160	20.2
No	632	79.8
Mothers’ watching TV		
Irregular	326	41.2
Regular	466	58.8
Mothers’ Facebook use		
Yes	63	8.0
No	729	92.0

* Adolescent girls’ parents’ family size.

**Table 2:** SRH knowledge-related items of older adolescent girls.

Items	Definitely True/Probably True *N* (%)	Not Sure *N* (%)	Definitely False/Probably False *N* (%)	Mean (95% CI)
Only physical change but no psychological change occurs during adolescence	248 (31.3)	53 (6.7)	491 (62.0)	3.65 (3.55–3.76)
Menstruation is one form of disease	442 (55.8)	65 (8.2)	285 (36.0)	2.67 (2.53–2.78)
The menstrual cycle more than once within a month is not a problem	101 (12.8)	109 (13.8)	582 (73.5)	4.20 (4.12–4.30)
A couple could risk an unwanted pregnancy if they do not follow any method of family planning	522 (65.9)	221 (27.9)	49 (6.2)	4.16 (4.08–4.24)
Taking birth control has no adverse effect on the sexual relationship of a couple	238 (30.1)	491 (62.0)	63 (8.0)	3.41 (3.34–3.49)
A birth to the conception interval of at least two years can reduce the risk of adverse maternal health	439 (55.4)	266 (33.6)	87 (11.0)	3.82 (3.73–3.91)
Unintended or unplanned pregnancy might cause for abortion	329 (41.5)	442 (55.8)	21 (2.7)	3.69 (3.62–3.76)
The carrier of the STIs may unintentionally transmit the virus to its partner	434 (54.8)	335 (42.3)	23 (2.9)	3.95 (3.87–4.02)
HIV spreads through the mosquito and flea	410 (51.8)	65 (8.2)	317 (40.0)	2.82 (2.69–2.95)
HIV does not spread the virus from an infected person’s coughing and sneezing	201 (25.4)	93 (11.7)	498 (62.9)	2.24 (2.12–2.36)

**Table 3:** SRH attitude-related items of older adolescent girls.

Items	Strongly Agree/Agree *N* (%)	Neutral *N* (%)	Strongly Disagree/Disagree *N* (%)	Mean (95% CI)
Sexual education leads to more sex	252 (31.8)	261 (33.0)	279 (35.2)	3.17 (3.08–3.27)
The school’s textbook lacks sufficient knowledge concerning SRH	514 (64.9)	120 (15.2)	158 (19.9)	3.66 (3.57–3.75)
The school teaching system is insufficient about SRH	484 (61.1)	109 (13.8)	199 (25.1)	3.54 (3.44–3.63)
A teenage girl does not go into the kitchen during her menstrual cycle	75 (9.5)	122 (15.4)	595 (75.1)	4.29 (4.21–4.37)
During the menstrual cycle, an adolescent girl should not touch anyone	72 (9.1)	97 (12.2)	623 (78.7)	4.39 (4.31–4.47)
An adolescent girl during the menstrual period should not go outside and to college	109 (13.7)	74 (9.3)	609 (76.9)	4.24 (4.15–4.33)
A woman ought not to brush her hair during her menstrual cycle	64 (8.1)	86 (10.9)	642 (81.1)	4.46 (4.38–4.54)
An adolescent girl should not look in the mirror during her menstrual period	54 (6.8)	85 (10.7)	653 (82.4)	4.48 (4.40–4.55)
Anyone could sin if s/he adopts the birth control method	59 (7.4)	118 (14.9)	615 (77.7)	4.35 (4.27–4.43)
Anyone who receives STIs should cover it up	64 (8.1)	101 (12.8)	627 (79.2)	4.42 (4.33–4.49)

**Table 4:** SRH practice-related items of older adolescent girls.

Items	Always/Often *N* (%)	Sometimes *N* (%)	Rarely/Never *N* (%)	Mean (95% CI)
I want to learn more new SRH details	413 (52.1)	271 (34.2)	108 (13.6)	3.74 (3.65–3.83)
I try to keep my SRH issues secret	116 (14.6)	169 (21.3)	507 (64.0)	3.99 (3.90–4.09)
I feel timid and afraid of my adolescent physical and psychological changes	171 (21.6)	211 (26.6)	410 (51.8)	3.57 (3.42–3.67)
I use cloth during menstrual periods instead of the sanitary pad	309 (39.0)	186 (23.5)	297 (37.5)	2.99 (2.89–3.12)
I keep my mother or older sister updated when I am menstruating	499 (63.0)	183 (23.1)	110 (13.9)	3.93 (3.83–4.02)
I change the pad or clean cloth after 5–6 h during the menstrual period	587 (74.1)	68 (8.6)	137 (17.3)	4.06 (3.96–4.16)
I eat more nutritious food during menstrual cycles	559 (70.6)	134 (16.9)	99 (12.5)	4.05 (3.95–4.13)
I do my daily activities during the menstruation time	625 (78.9)	118 (14.9)	49 (6.2)	4.36 (4.29–4.44)
I feel at ease when talking about SRH	484 (61.1)	29 (37.0)	279 (35.2)	3.60 (3.47–3.72)
Using the phrase ‘I am sick’ is comfortable for me during menstrual cycles	456 (57.6)	133 (16.8)	203 (25.6)	2.43 (2.32–2.54)

**Table 5:** Factors influencing SRH-related knowledge, attitude, and practice of older adolescent girls.

Variable	Knowledge on SRH			Attitude toward SRH			Practice of SRH		
	β	*t*	*p*	β	*t*	*p*	β	*t*	*p*
Constant		37.93	<0.001		42.66	<0.001		35.87	<0.001
Group (non-science vs. science)	0.29	7.99	<0.001	0.17	4.62	<0.001	0.07	1.98	0.048
Area of residence (rural vs. urban)	0.04	0.94	0.349	0.20	4.93	<0.001	0.22	5.39	<0.001
Watching TV (irregular vs. regular)	0.03	0.92	0.356	0.01	0.40	0.692	0.05	1.54	0.125
Mobile use (no vs. yes)	−0.00	−0.12	0.908	0.02	0.49	0.625	−0.02	−0.65	0.513
Fathers’ education (up to secondary vs. above secondary)	0.03	0.84	0.401	−0.00	−0.04	0.971	0.01	0.19	0.848
Mothers’ education (up to secondary vs. above secondary)	0.01	0.33	0.743	0.00	0.09	0.926	0.04	1.08	0.282
Mothers’ watching TV (irregular vs. regular)	0.02	0.43	0.667	0.02	0.59	0.557	0.08	2.27	0.024
Frequency of SRH discussion (irregular vs. regular)	0.04	1.31	0.192	0.10	2.95	0.003	0.09	2.82	0.005
Knowledge on period (after menarche vs. before menarche)	0.05	1.54	0.123	0.07	2.06	0.040	0.12	3.72	<0.001
Source of SRH knowledge (others vs. mother)	−0.02	−0.52	0.605	−0.04	−1.14	0.254	0.08	2.52	0.012
Ever read about or watched any SRH content on media (no vs. yes)	0.21	6.50	<0.001	0.22	6.54	<0.001	0.18	5.49	<0.001
* Ever talked with a doctor or health worker about SRH (no vs. yes)				−0.04	−1.07	0.287	0.08	2.42	0.016
	*R*^2^ = 0.19	*F* = 17.42	<0.001	*R*^2^ = 0.21	*F* = 16.76	<0.001	*R*^2^ = 0.22	*F* = 18.55	<0.001

Note: * The variable was not included for multiple linear regression predicting the level of knowledge on reproductive health as the *p* values were >0.10 in bivariate analyses.

## Abbreviations

AIDS: Acquired immunodeficiency syndrome; ANOVA: Analysis of variance; BBS: Bangladesh Bureau of Statistics; BDHS: Bangladesh Demographic and Health Survey; BIED: BRAC Institute of Educational Development; CI: Confidence interval; HIV: Human immunodeficiency virus; KAP: Knowledge, attitude, and practice; MOHFW: Ministry of Health and Family Welfare; NIPORT: National Institute of Population Research and Training; SD: Standard deviation; SDGs: Sustainable Development Goals; SRH: Sexual and reproductive health; SPSS: SPSS: Statistical package for the social sciences; STIs: Sexually transmitted infections; UNFPA: United Nations Population Fund; UNICEF: United Nations Children’s Fund; VAW: Violence against Women; WHO: World Health Organization.
